# Using Mobile Health Intervention to Improve Secondary Prevention of Coronary Heart Diseases in China: Mixed-Methods Feasibility Study

**DOI:** 10.2196/mhealth.7849

**Published:** 2018-01-25

**Authors:** Shu Chen, Enying Gong, Dhruv S Kazi, Ann B Gates, Rong Bai, Hua Fu, Weixia Peng, Ginny De La Cruz, Lei Chen, Xianxia Liu, Qingjie Su, Nicolas Girerd, Kamilu M Karaye, Khalid F Alhabib, Lijing L Yan, JD Schwalm

**Affiliations:** ^1^ Global Health Research Center Duke Kunshan University Kunshan China; ^2^ Department of Medicine (Cardiology) University of California, San Francisco San Francisco, CA United States; ^3^ Department of Epidemiology and Biostatistics University of California, San Francisco San Francisco, CA United States; ^4^ Division of Cardiology Zuckerberg San Francisco General Hospital San Francisco, CA United States; ^5^ Exercise Works Ltd. Derby United Kingdom; ^6^ National Clinical Research Center for Cardiovascular Diseases Beijing China; ^7^ Department of Cardiology Beijing Anzhen Hospital Capital Medical University Beijing China; ^8^ School of Public Health Fudan University Shanghai China; ^9^ Global Health Research Center Duke Kunshan University Kunshan China; ^10^ Department of Cardiology Hainan Provincial Nongken General Hospital Haikou China; ^11^ Department of Neurology Hainan Provincial Nongken General Hospital Haikou China; ^12^ Institut Lorrain du Coeur et des Vaisseaux Louis Mathieu Centre d’Investigations Cliniques Centre Hospitalier Universitaire de Nancy Nancy France; ^13^ Cardiovascular and Renal Clinical Trialists French Clinical Research Infrastructure Network Nancy France; ^14^ Department of Medicine Bayero University/Aminu Kano Teaching Hospital Kano Nigeria; ^15^ Department of Cardiac Sciences King Fahad Cardiac Center, College of Medicine King Saud University Riyadh Saudi Arabia; ^16^ Duke Global Health Institute Duke University Durham, NC United States; ^17^ Population Health Research Institute Hamilton Health Sciences Hamilton, ON Canada; ^18^ McMaster University Hamilton, ON Canada

**Keywords:** coronary heart disease, secondary prevention, medication adherence, mobile applications, text messaging

## Abstract

**Background:**

Coronary heart disease (CHD) is the leading cause of cardiovascular mortality worldwide, yet implementation of evidence-based strategies for secondary prevention remains suboptimal.

**Objective:**

This study aimed to evaluate the feasibility, specifically the usability and acceptability, and estimate the preliminary effectiveness of a mobile health (mHealth) intervention targeting both physicians and patients to improve adherence to evidence-based medications and lifestyle modifications.

**Methods:**

We conducted a 12-week pre-post interventional pilot study at two sites in Shanghai and Hainan, China. Physicians used the app designed in this study to prescribe evidence-based medicines and record patient information. Eligible and consenting patients received automatic text messages or voice calls 4 to 5 times per week for 12 weeks on medication adherence and healthy behaviors. Interviews were conducted among 10 physicians and 24 patients at the two sites for their thoughts on medication adherence and feedback on the usability and acceptability. Questions on usability and acceptability were also asked in a patient follow-up survey. With regard to estimating effectiveness, the primary outcome was medication adherence (as estimated by the Morisky Green Levine Scale) at 12 weeks. Secondary outcomes included physical activity, smoking status, fruits and vegetables consumption, and facility visit frequency.

**Results:**

Interview findings and patient survey showed the good usability and acceptability of the intervention. Among 190 patients who completed the intervention, there was a significant increase in medication adherence (odds ratio [OR] 1.80, 95% CI 1.14-2.85). The study also showed decrease of smokers’ percentage (−5%, *P*=.05), increase of daily vegetables consumption frequency (+0.3/day, *P*=.01), and community health care center visit frequency (+3 in 3 months, *P*=.04). The following site-specific differences were noted: medication adherence appeared to increase in Hainan (OR 14.68, 95% CI 5.20-41.45) but not in Shanghai (OR 0.61, 95% CI 0.33-1.12).

**Conclusions:**

Our study demonstrated that the intervention was feasible in both a tertiary care center and an urban community health center in China. Preliminary results from pre-post comparison suggest the possibility that provider and patient-linked mHealth interventions may improve medication adherence and lifestyle modifications among CHD patients, especially in resource-scarce settings. Randomized controlled trials are needed to verify the findings.

## Introduction

### Background

Coronary heart disease (CHD) is the leading cause of cardiovascular mortality worldwide [1]. Despite a recent decline in high-income countries [2,3], CHD mortality continues to rise rapidly in low- and middle-income countries [4]. In China, CHD mortality was approximately 100 per 100,000 person in 2013, ranking second only to stroke among causes of cardiovascular deaths [5,6].

Clinical guidelines recommend a combined strategy of using evidence-based medicine and lifestyle modifications for secondary prevention. However, the implementation of these recommendations is suboptimal. The Prospective Urban Rural Epidemiology (PURE) study found that more than 50% of community-based patients from 17 high-, middle-, and low-income countries with known cardiovascular diseases did not take any medicines recommended by guidelines [7]. Even when patients have been initiated on evidence-based medicines at hospital discharge, the medication adherence is usually poor regardless of the socioeconomic status of the patients [8-10]. Evidence from a large multicenter retrospective analysis among more than 2901 CHD patients in China showed that less than 10% of patients used aspirin, clopidogrel, Angiotensin converting enzyme (ACE) inhibitors, or calcium antagonist at 1 year of hospital discharge [8]. Patients were also observed to have a high prevalence of residual lifestyle risk factors [9]. For example, 70% of men and 8% of women with CHD were active smokers in a large Chinese multicenter cross-sectional survey [9].

Addressing the implementation challenges of secondary prevention of CHD requires feasible, scalable, and cost-effective solutions. Recent advances in the widespread use of mobile phones and technology have made mobile health (mHealth) a promising solution. Short message service (SMS) for text messaging is one of the mHealth approaches that has been used in the management of diseases [10] such as asthma [11], human immunodeficiency syndrome (HIV) [12,13], malaria [14], diabetes [15], hypertension [10], and CHD [16,17]. However, the evidence of the effect of mHealth on medication adherence, diet, and physical activities remains inconclusive [10]. Furthermore, few studies have examined multifaceted interventions targeting both health care providers and patients for the improved delivery of secondary prevention of CHD [16].

### Information for the Adherence and Knowledge Exchange Heart Disease Medicines Study

We designed and implemented the Adherence and Knowledge Exchange heart disease medicines study (TAKEmeds study) to examine whether an mHealth intervention can improve the patient adherence to evidence-based medications for the secondary prevention of CHD. The intervention includes a provider-facing mobile app guiding medicine prescription and a patient-directed text message or voice call system that promotes medication adherence and behavior modification to optimize secondary prevention of CHD. The specific purpose of this study was to pilot the intervention in China to examine its feasibility, including usability, acceptability, and preliminary effectiveness. This study follows the mHealth evidence reporting and assessment checklist [18].

## Methods

### Study Design

**Figure 1:**
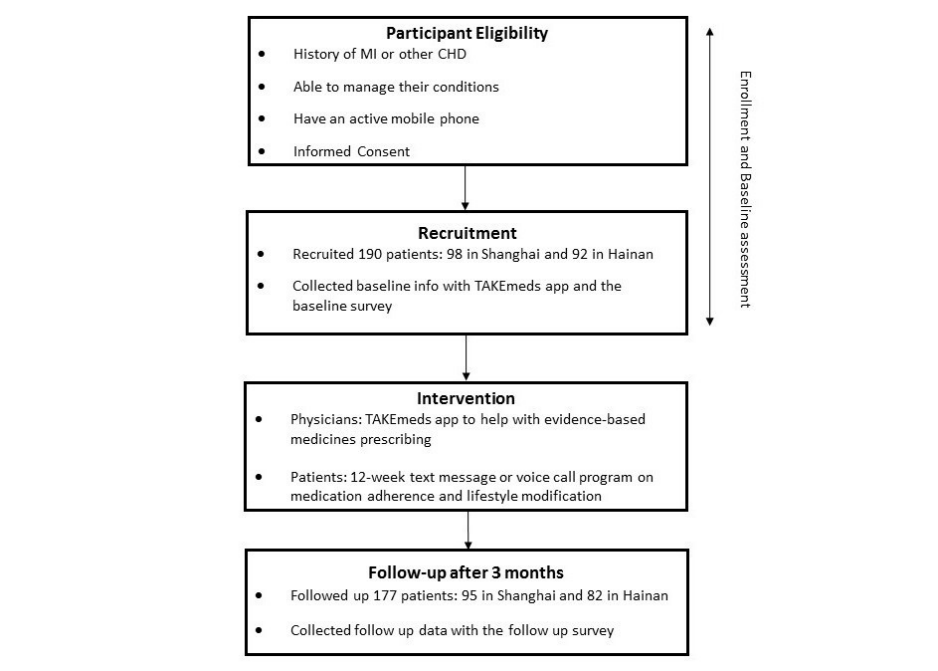
The Adherence and Knowledge Exchange heart disease medicines (TAKEmeds) study flowchart.

The TAKEmeds project was a pre-post multicenter interventional pilot study conducted in a community health care center in Shanghai and a tertiary hospital in Hainan Province, China (see Figure 1 for the flowchart of the study). The study was conceived and developed as a part of the World Heart Federation’s Emerging Leader program. Implementation in China was led by the Global Health Research Center, Duke Kunshan University, in local partnership with the School of Public Health, Fudan University, and Hainan Provincial Nongken General Hospital (HPNGH). The study obtained ethical approval from the institutional review boards at Duke University Health System, Fudan University, and HPNGH. The trial was registered in the clinicaltrials.gov database on November 2015 (NCT02597205). The design process of the intervention tools has been previously reported [17].

### Study Sites

The TAKEmeds study was conducted in a community health clinic in Longhua Street, Shanghai, and a tertiary care facility in Haikou, Hainan. Shanghai is a large metropolis with around 23 million people and the third highest gross domestic product (GDP) per capita in China, whereas Hainan is a tropical island province in southern China with around 8 million people, ranked 21st out of 34 provinces in terms of GDP per capita [19]. The number of physicians per 1000 population in Shanghai was 2.17 versus 1.38 in Hainan in 2012 [20]. The Longhua Street Community Health Center in Shanghai serves 74,827 populations in 20,789 families. The Hainan Provincial Nongken General Hospital is one of the two tertiary hospitals serving Haikou, the capital city in Hainan, with around 1800 beds serving 590,300 residents. These two sites were selected because of their following distinctive features: primary health care center in a developed area and tertiary hospital in a developing area, which aids generalizability of our findings to disparate health systems across China.

### Study Participants

Patients were eligible if (1) they had a history of a myocardial infarction (MI) or obstructive CHD (as clinically diagnosed by the treating physician), (2) they were physically and mentally able to manage their health care themselves, (3) they owned a mobile phone and were comfortable with receiving messages, (4) they were able and willing to provide informed consent, and (5) they were above 18 years of age. Patients who refused consent, participated in another study, or were severally ill with less than 3 months of expected survival were excluded.

In the community health center in Shanghai, patients were identified through the CHD patient list by general practitioners. In the tertiary hospital in Hainan, patients were identified through screening daily admissions and from cardiology outpatient clinic visits. The clinic was affiliated with the tertiary hospital. A promotion flyer that described the study aim and intervention and provided information about how to join the study was distributed to patients for recruitment. Patients were remunerated at the beginning and at end of study with a small gift (combined value 80 Ren Min Bi [RMB] [US $13]).

Physicians were eligible if (1) they owned an Android mobile phone, (2) they took care of coronary artery diseases patients, and (3) they were willing to provide informed consent. Physicians that chose to participate in intervention were given a monetary compensation of around 200 RMB (around US $32) in total.

### Intervention

The technology-enabled multifaceted intervention was developed to improve patient adherence to medications and modify their lifestyles. At the provider level, we developed an Android-based mobile app, named TAKEmeds, to support prescription of evidence-based medicines and facilitate patient recruitment. At the patient level, we developed a message bank with 60 messages that could be automatically sent to patients through a central server, nonrepetitively, over 12 weeks. These messages were based on current international guideline recommendations and evidence promoting medication adherence and lifestyle modifications. Patients who were active cigarette smokers at the time of enrollment also received tailored messages that help support smoking cessation. The TAKEmeds app and short messages were beta-tested and validated before the study rolled out. The app test version was validated by researchers and physicians, and the messages were tested among the employees in the development company. Details of app and messages development using principles of user-centered design have been described elsewhere [17].

**Figure 2:**
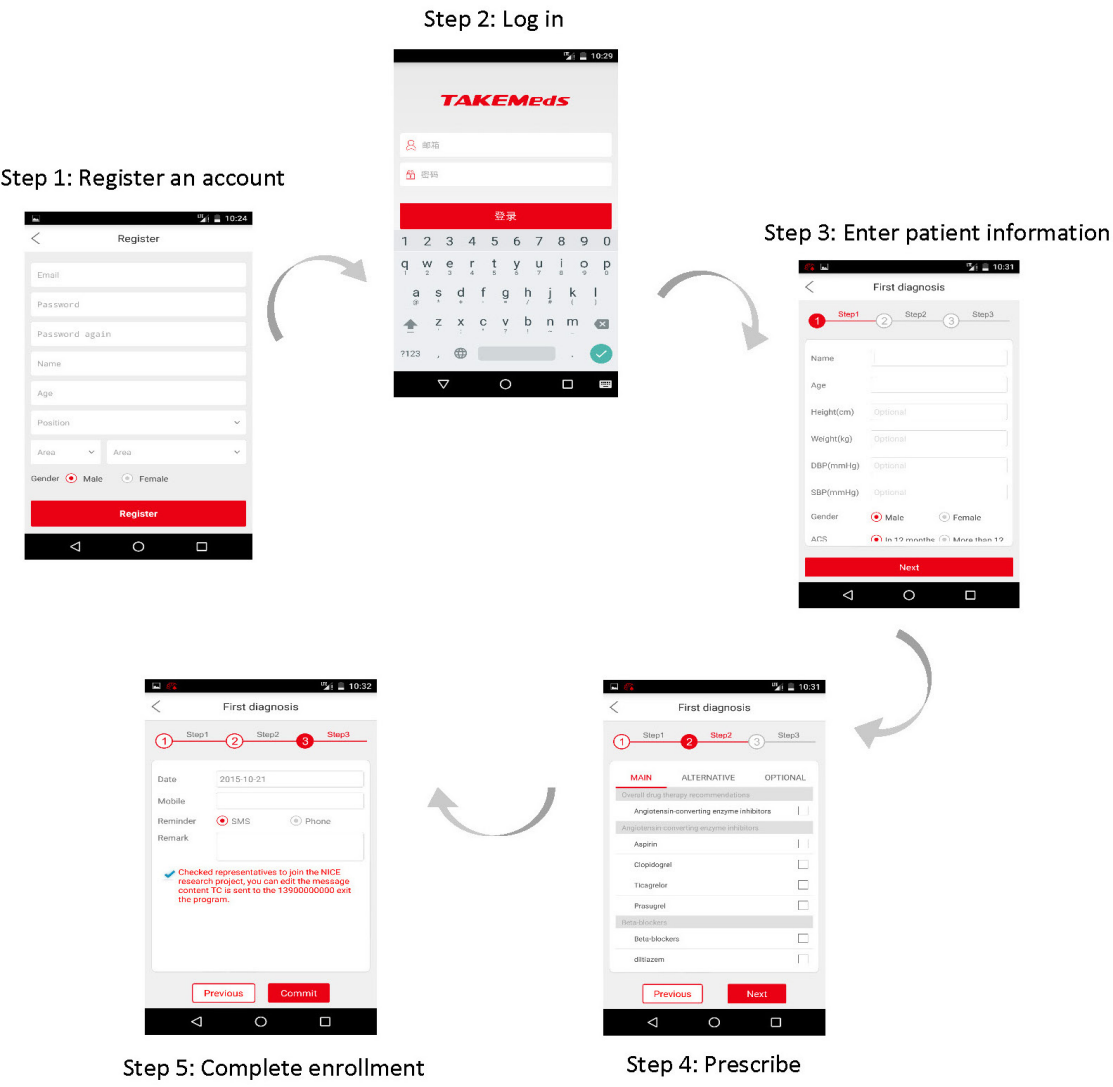
Flowchart of the Adherence and Knowledge Exchange heart disease medicines (TAKEmeds) app operational procedures.

After a brief training session, physicians used the app to record basic demographic and clinical information about eligible patients as well as to prescribe medications for secondary prevention of CHD. The TAKEmeds app made medication recommendations based on the guidelines for secondary prevention of MI published by the UK’s National Institute for Health and Care Excellence (NICE) [21]. The NICE guideline was chosen as it was widely accepted and adopted internationally, and there are currently no national guidelines for MI secondary prevention in China. We also consulted a cardiologist in Beijing Anzhen Hospital (one of the coauthors) on its adaption in China before it was adopted. Physicians were able to choose medicines from the recommendations on the app after they entered time interval since heart disease onset (≥or <1 year). The app also provided information for physicians such as an overview of treatment options for MI patients with comorbidities and the importance of lifestyle changes. The flowchart of the app operational procedures and screenshots are presented in Figure 2, and more details can be found in the published development paper elsewhere [17].

**Figure 3:**
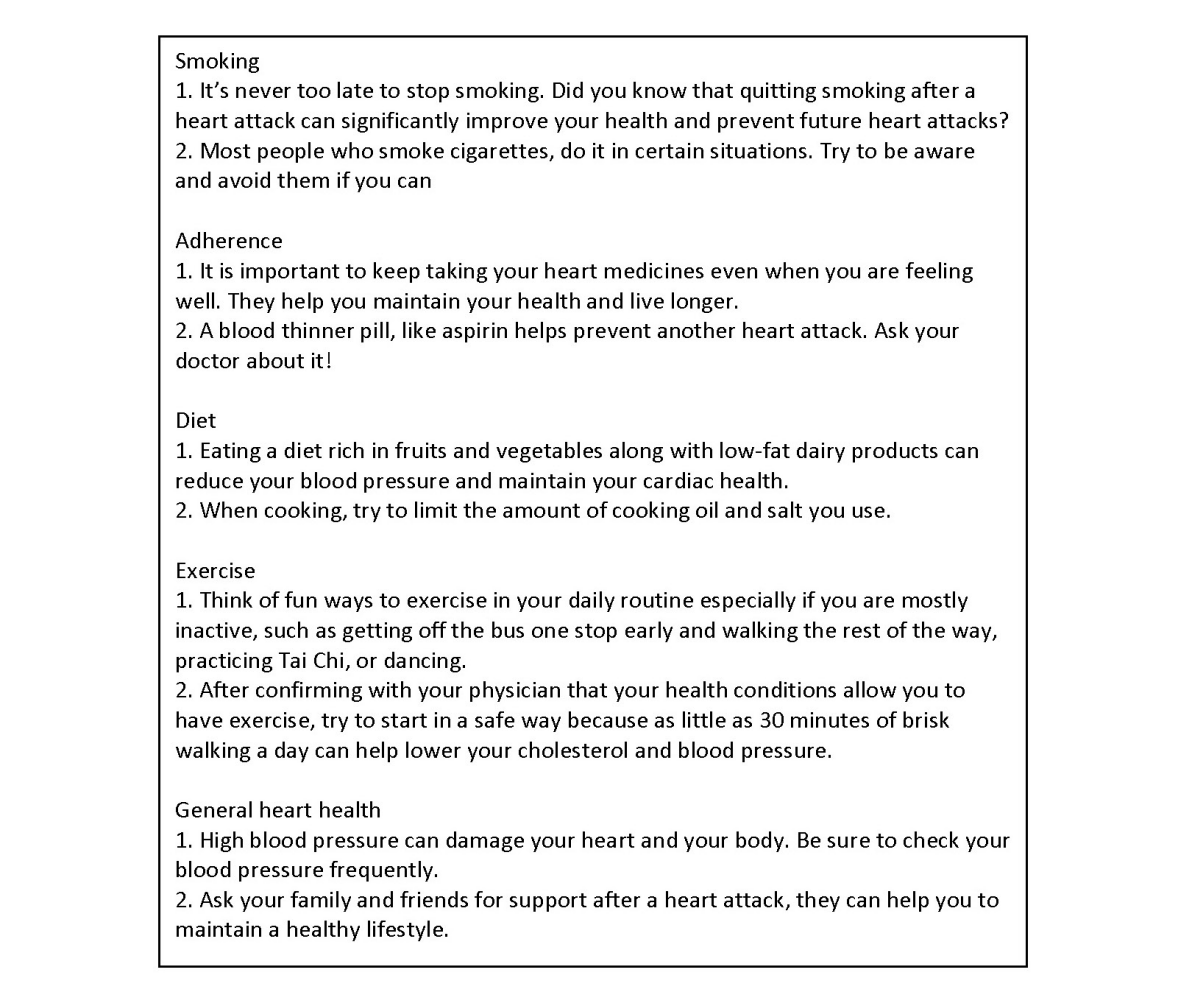
Examples of text messages developed and used in the Adherence and Knowledge Exchange heart disease medicines (TAKEmeds) study.

At the time of enrollment, patients could choose to receive messages as short text messages or recorded phone calls. The phone calls were provided as an option for senior patients not comfortable with text messages, and they were machine recorded and shared the same contents (details of the messages and phone calls can be found in the paper published elsewhere) [17]. Messages covered the following 5 modules: medication adherence, physical activity, diet, smoking cessation, and general heart health. Example messages of the 5 modules are presented in Figure 3. We set the algorithm to send 4 to 5 messages per week to patients during weekdays for 12 weeks. Nonsmokers received one medication adherence message, one nutrition message, one exercise message, and one general heart health message on 4 random weekdays. Smokers received one additional message on smoking cessations. These messages were sent at one of the four random time slots: 9:00-9:10 AM, 12:00-12:10 PM, 3:00-3:10 PM, or 5:00-5:10 PM on the weekdays. As a part of the enrollment process, patients were also told the messages were one-way only (ie, patients could not respond to the messages with queries), and they could opt to stop receiving messages at any time by either informing their physicians or calling a given number.

### Outcome Measures

The primary outcome was change in proportions of patients with different medication adherence status at 12 weeks compared with baseline. Medication adherence was measured by the 4-item Morisky Green Levine Scale [22]. Patients were classified into following 3 categories of adherence: low (score 3-4), intermediate (score 1-2), and high (score 0) [22].

Secondary outcomes included the following: (1) change in proportions of patients with different physical activity level, measured by the short-form version of the International Physical Activity Questionnaire [23]; (2) change in proportions of patients with different smoking status (classified as smoking every day, smoking occasionally, and never smoking); (3) change in the median of frequencies of patients consuming vegetables and fruits, calculated with the 6-item brief dietary assessment tool from the Behavioral Risk Factor Surveillance System fruit and vegetable dietary intake module [24]; and (4) change in the frequencies of patients visiting health facilities over the 3 months.

We conducted a patient survey at the end of the study to assess patient satisfaction toward the intervention, patient perceptions regarding the helpfulness of the intervention, and feedback regarding how they would prefer to receive messages in the future. To assess the affordability of the intervention, we collected basic costs of the messages or voice calls based on the charges of the carrier. We did not collect all costs incurred in this study, as it was not our intention to conduct a full economic analysis.

### Sample Size Estimates

Sample size for this pilot study was primarily determined by budget and feasibility. The calculations were also done based on the primary outcome of medical adherence change at 12 weeks. Assuming the odds for patients with high medication adherence versus middle and low medication adherence (or high and middle medication adherence vs low medication adherence) were 10 times larger after the intervention, a total of 74 patients would have 80% power (two-sided alpha of .05). Allowing for 10% loss to follow-up and multiple sites, a total of 166 patients (83 patients in each site) would provide 80% power to detect the difference.

### Data Collection and Analysis

#### Quantitative Data

We collected patient quantitative data from two sources. The first was the self-reported survey at baseline and 12 weeks at follow-up. The survey was administered by trained researchers in Shanghai and resident physicians in Hainan, and they collected data on primary and secondary outcome measures. In Shanghai, trained researchers visited the primary health care centers at baseline and follow-up to collect the data. The second was the TAKEmeds app into which demographic and clinical history were entered by physicians at the time of enrollment.

In addition, we estimated communication costs based on the recorded number of messages and the estimated duration of voice calls. The pre-post paired analyses were done at the individual patient level. As for the primary outcome, we built an ordinal (ordered) logistic regression model to test the intervention effect on medication adherence, and both univariate and covariate analyses were conducted. Time (before or after the intervention) was set as the dummy variable. Covariates in the model include age, gender, region, heart disease type, heart disease diagnosis time (≤ or >1 year), hypertension, diabetes status, and number of medications taken. Region was further treated as an interaction term to assess the outcome differences at the two sites. As for secondary outcomes, we used Wilcoxon test to compare the before and after change for physical activity, smoking status, fruits and vegetables consumption, and center visit frequency, and subgroup analysis was conducted. Analyses were conducted using STATA version 13.0 (StataCorp, College Station, TX, USA). All statistical tests were two-tailed with significance level set at .05.

#### Qualitative Data

The qualitative data were collected through process evaluations at the middle and end of the study. Two focus group discussions were conducted among physicians in Shanghai (n=4) and Hainan (n=6), each lasting around 1 hour. Individual interviews were conducted among randomly selected patients (n=24, 12 from each site) at both sites, each lasting 20 min. The qualitative component was to understand the perceived barriers to improving medication adherence and their feedback on the usability and acceptability of the intervention.

Interviews and focus group discussions were recorded and transcribed. We used the thematic framework to analyze the qualitative results by using the NVivo version 10.0 qualitative data analysis software (QSR International Pty Ltd). Specifically, we coded the transcripts into different nodes of meaning and then grouped the nodes with similar meaning under one theme.

## Results

Patient recruitment occurred from May 2015 to August 2015, and follow-up was completed by November 2015. A total of 190 patients were enrolled by 10 physicians, with 98 patients in Shanghai and 92 patients in Hainan. We successfully followed up 177 patients (follow-up rate 93.2%) after the 12-week intervention. Loss to follow-up rate was 3.1% (n=3) in Shanghai and 10.9% (n=10) in Hainan (*P*=.03). No significant differences were observed between the profiles of the loss-to-follow patients compared with followed patients at the two sites combined or at each site. The results section presented findings of the preliminary effectiveness, usability, and acceptability of the intervention.

### Baseline Characteristics

Baseline demographics, cardiac history, and medications of the 190 patients are presented in Table 1. Patients were 31.6% female, with a mean age of 67 years (SD 10). Patients in Hainan were 5 years younger than those in Shanghai (64 years vs 69 years, *P*=.004) with more severe CHD (a significantly higher proportion had a history of MI, and a higher proportion had been diagnosed with CHD within 1 year). All patients in Hainan took at least 2 cardiac medicines, whereas 79.6% of patients in Shanghai did so. However, fewer patients in Hainan had been previously diagnosed with hypertension (47.8% vs 71.4% in Shanghai). The vast majority of patients (94.2%) chose to receive the information via text messages rather than via phone calls.

The mean age was 62 years (SD 10) for interviewed patients. On average, patients in Hainan (mean age: 57 years, SD 12) were younger than those in Shanghai (mean age: 68 years, SD 8). The percentage of male patients at both sites was 75.0%. Six physicians in Hainan (all cardiologists) and 4 physicians in Shanghai (all general practitioners) participated in this study and focus group discussions. The mean age of physicians was 36 years (SD 5). Physicians in Shanghai (mean age 35 years, SD 4) were generally younger than those in Hainan (mean age: 37 years, SD 6); at both sites, 50.0% of the physicians were males. All Shanghai physicians were in practice for ≤10 years, whereas Hainan physicians were in practice between 11 and 20 years.

**Table 1:** Demographic, health status, and medicine use of patients at baseline.

Baseline characteristics		Total (N=190)	Shanghai (N=98)	Hainan (N=92)	*P* value
**Gender, n (%)**					
	Female	60 (31.6)	35 (35.7)	25 (27.2)	.21
	Male	130 (68.4)	63 (64.3)	67 (72.8)	
Age in years, mean (SD)		67 (10)	69 (8)	64 (12)	.004
Diagnosed with hypertension, n (%)		114 (60.0)	70 (71.4)	44 (47.8)	.001
Diagnosed with diabetes, n (%)		49 (25.8)	28 (28.6)	21 (22.8)	.34
**Heart disease type, n (%)**					
	MI^a^	105 (55.3)	47 (48.0)	58 (63.0)	.03
	Non-MI	85 (44.7)	51 (52.0)	34 (37.0)	
**Time of diagnosis of heart disease, n (%)**					
	Within 1 year	67 (35.3)	17 (17.3)	50 (54.3)	<.001
	More than 1 year	123 (64.7)	81 (82.7)	42 (45.7)	
**Number of cardiac medications, n (%)**					
	1	20 (10.5)	20 (20.4)	0	
	2	37 (19.5)	26 (26.5)	11 (12.0)	<.001
	3 and more	133 (70.0)	52 (53.1)	81 (88.0)	
**Medication class, n (%)**					
	ACE^b^ inhibitors and ARB^c^	43 (22.6)	27 (27.6)	16 (17.4)	.09
	Antiplatelets	152(80.0)	67 (68.4)	85 (92.4)	<.001
	Beta-blockers	118 (62.1)	52 (53.1)	66 (71.7)	.01
	Aldosterone antagonists	9 (4.7)	0	9 (9.8)	.02
	Statins and other lipid-lowering agents	131 (68.9)	59 (60.2)	72 (78.3)	.01

^a^MI: myocardial infarction.

^b^ACE inhibitors: angiotensin-converting enzyme inhibitors.

^c^ARB: angiotensin receptor blockers.

**Table 2:** Effect of the Adherence and Knowledge Exchange heart disease medicines (TAKEmeds) intervention on the primary outcome: medication adherence.

Medication adherence		Baseline	Follow-up	Unadjusted OR^a^ (95% CI)	*P* value	Adjusted^b^ OR (95% CI)	*P* value
**Combined, n (%)**							
	High	107 (61.5)	121 (69.5)	1.74 (1.09-2.78)	.02	1.80 (1.14-2.85)	.01
	Middle	44 (25.3)	44 (25.3)				
	Low	23 (13.2)	9 (5.2)				
**Shanghai, n (%)**							
	High	61 (64.9)	47 (50.0)	0.71 (0.42-1.22)	.22	0.61 (0.33-1.12)	.11
	Middle	19 (20.2)	38 (40.4)				
	Low	14 (14.9)	9 (9.6)				
**Hainan, n (%)**							
	High	46 (57.5)	74 (92.5)	12.85 (4.62-35.76)	<.001	14.68 (5.20-41.45)	<.001
	Middle	25 (31.3)	6 (7.5)				
	Low	9 (11.3)	0				

^a^OR: Odds ratio.

^b^Adjusted for age, gender, region, heart disease type, heart disease diagnosis time (< or >1 year), hypertension, diabetes status, and number of medications taken.

### Preliminary Estimate of Effectiveness: Primary Outcome

#### Quantitative Results

The primary outcome results are presented in Table 2. We observed a significant improvement in medication adherence in participants postintervention, either before (odds ratio [OR] 1.74, *P*=.02) or after adjusting for the covariates (OR 1.80, *P*=.01). The percentage of participants reported with high medication adherence increased from 61.5% to 69.5% and with low medication adherence decreased from 13.2% to 5.2%. Participants who took 2 (OR 3.11, *P*=.02) or more than or equal to 3 medicines (OR 2.66, *P*=.03) tended to improve their medication adherence more significantly, compared with those who only took 1 medicine, after adjusting the covariates. Results of the full model can be found in Multimedia Appendix 1.

We identified a significant interaction between location of inclusion and intervention effect on medication adherence (*P*<.001). Results of the model with region as the interaction term can be found in Multimedia Appendix 1. The unadjusted and adjusted outcomes were presented in Table 2 (descriptions here were adjusted outcomes only). In Hainan, there was a significant improvement in medication adherence (OR 14.68, *P*<.001), meaning that after the intervention, the odds for patients in Hainan with high medication adherence versus middle and low medication adherence (or high and middle medication adherence vs low medication adherence) were 14.68 times larger, given the other variables were held constant. This could be shown in the increase of percentage of participants with high medication adherence from 57.5% to 92.5%. In contrast, we did not observe significant change in medication adherence in patients included in Shanghai (OR 0.61, *P*=.11).

#### Qualitative Results

The qualitative analysis of the patient and physician interviews revealed several factors that might lead to suboptimal medication adherence. Factors arising from the current flawed health system include the following: first, there existed no standard patient follow-up scheme, especially for cardiovascular patients. One physician stated the following:

There are follow-up interviews of hypertension and diabetes patients, while none for cardiovascular patients.

Physician, Shanghai, female, 33 years old

Another physician stated the following:

It is our system’s problem. There’s no integrated NCD management scheme to follow-up with patients.

Physician, Hainan, male, 35 years old

Second, facilities suffered shortage of medicine supplies. One physician stated the following:

There are some other medicines. Although they are cheap, and we want to use them, there are no supplies. There’s no on the market, and pharmaceutical companies are unwilling to produce

Physician, Hainan, female, 38 years old

Another physician stated the following:

It is difficult to prescribemedicines because community hospitals do not have some essential medicines for myocardial infarction patients. We lost them to tertiary hospitals.

Physician, Shanghai, female, 33 years old

One physician stated the following:

For example, the community hospital only has short-acting Betaloc, instead of long-acting one. And other medicines are the same. It would be best if I did not have to go to large hospitals, but I have no other way.

Patient, Shanghai, female, 80 years old

Third, the intense patient-physician relationship reduced patients’ trust in physicians. One physician stated the following:

Patients would come only for prescriptions. They would think that community centers do not have good solutions. So it is hard to manage their conditions.

Physician, Shanghai, male, 30 years old

Another physician stated the following:

There’s a crisis of trustiness. There are a lot of people think, we are aiming at something when we ask patient to take medicines.

Physician, Hainan, female, 38 years old

Fourth, the unaffordability of the medicines kept patients from adhering to physicians. One physician stated the following:

I don’t have much income as I have retired. It may be difficult, but there’s no stipend for me.

Patient, Shanghai, male, 68 years old

Another physician stated the following:

The patients may find the medicines are expensive and they cannot afford. So they have to quit it

Physician, Hainan, female, 38 years old

Apart from factors attributing to current health system, another reason was that the prevention awareness among the patients was low. One physician stated the following:

Patients would only come and see a doctor when symptoms appear.

Physician, Shanghai, female, 33 years old

In addition, medicines’ side effects were another downside factor for patients to adhere.

### Preliminary Estimate of Effectiveness: Secondary Outcomes

#### Quantitative Results

The secondary outcomes are presented in Table 3. We found an improving trend in patients’ smoking status: the percentage of patients that reported no smoking increased from 83.0% to 87.5% (*P*=.05). We observed an increase in the daily frequency of consuming vegetables (from 2.4 to 2.7/day, *P*=.01). The results showed an increase in community health care center visit frequency from 7 to 10 during the 12 weeks (*P*=.04) in Shanghai. We did not find significant changes among patients in terms of fruits consumption (*P*=.18) or physical activity (*P*=.91).

#### Qualitative Results

The interviews and focus group discussions collected physicians’ and patients’ feedback on whether and how the intervention could help modify lifestyles and enhance physician-patient communication.

**Table 3:** Paired pre-post comparison of secondary outcomes.

Secondary outcomes		Total			Shanghai			Hainan		
		Baseline	Follow-up	*P* value	Baseline	Follow-up	*P* value	Baseline	Follow-up	*P* value
**Physical activity, n (%)**										
	Highly active^a^	14 (8.0)	17 (9.7)	.91	7 (7.4)	8 (8.4)	.36	7 (8.6)	9 (11.1)	.57
	Minimally active^b^	112 (63.6)	105 (59.7)		69 (72.6)	62 (65.3)		43 (53.1)	43 (53.1)	
	Inactive^c^	50 (28.4)	54 (30.7)		19 (20.0)	25 (26.3)		31 (38.3)	29 (35.8)	
**Current smoking status, n (%)**										
	Everyday	18 (10.2)	13 (7.4)	.05	8 (8.4)	3 (3.2)	.06	10 (12.3)	10 (12.3)	.36
	Seldom	12 (6.8)	9 (5.1)		6 (6.3)	8 (8.4)		6 (7.4)	1 (1.2)	
	Never	146 (83.0)	154 (87.5)		81 (85.3)	84 (88.4)		65 (80.2)	70 (86.4)	
Fruits consumption, median frequency/ day		0.5	0.6	.18	0.6	0.8	.65	0.3	0.6	.17
Vegetable consumption, median frequency/day		2.4	2.7	.01	2.6	3.2	.001	1.8	2.2	.73
Facility visit, median frequency/3 months		3	3	.22	7	10	.04	1	2	.69

^a^Vigorous-intensity activity on at least 3 days, achieving a minimum of at least 1500 metabolic equivalent (MET)-minutes per week, OR 7 or more days of any combination of walking, moderate-intensity, or vigorous-intensity activities, achieving a minimum of at least 3000 MET-minutes per week.

^b^3 or more days of vigorous activity for at least 20 min per day OR 5 or more days of moderate-intensity activity or walking for at least 30 min per day OR 5 or more days of any combination of walking, moderate-intensity, or vigorous-intensity activities, achieving a minimum of at least 600 MET-min per week.

^c^Individuals who do not meet criteria for the above 2 categories are considered inactive.

The comments we received were generally positive, albeit a few negative reviews remained. One patient stated the following:

After I began to receive message, I eat less meat when I was asked, and I work out when I am asked.

Patient, Shanghai, female, 67 years old

Another patient stated the following:

It helps me receive more knowledge. For example, I received a message asked me to eat less meat. I don't like fish and I only eat meat. And the SMS asked me to eat less of it.

Patient, Shanghai, male, 74 years old

One physician claimed that it was hard to change patients’ lifestyle:

From my perspective, I would say that the effects are few. Because the lifestyle of patients is already mature, it is hard to be changed. And they don’t pay much attention to it.

Physician, Shanghai, male, 33 years old

Another physician stated that the intervention would take little effect in promoting communication with patients because they were too busy:

My patients are too many, and I don't have time to respond at all.

Physician, Hainan, male, 33 years old

### Usability and Acceptability

All interviewed patients in Shanghai and 10 out of the 12 interviewed patients in Hainan commented that the messages were easy to understand. All participated physicians commented that the app was easy to use; however, the use of prescription function was restricted. It took them 5 to 10 mins to set up a patient on the app.

**Table 4:** Perceived helpfulness of the intervention program and user feedback.

Characteristics		Total, n (%)	Shanghai, n (%)	Hainan, n (%)
**Helpful in**				
	Medication adherence (n=152)	123 (80.9)	68 (74.7)	55 (90.2)
	Healthy diet (n=173)	132 (76.3)	69 (75.8)	63 (76.8)
	Exercise (n=173)	115 (66.5)	53 (58.2)	62 (75.6)
	Smoking cessation (n=23)	14 (60.9)	3(30.0)	11 (84.6)
	Communication with physicians (n=173)	129 (74.6)	59 (64.8)	70 (85.4)
**Preferred way of receiving messages (n=171)**				
	Text messages	111 (64.9)	84 (92.3)	27 (33.8)
	Voice calls	57 (33.3)	4 (4.4)	53 (66.3)
	Other	3 (1.8)	3 (3.3)	0
**Preferred time of receiving messages (n=173)**				
	8:00-11:59 AM	73 (42.2)	40 (44.0)	33 (40.2)
	12:00-12:59 PM	3 (1.7)	2 (2.2)	1 (1.2)
	1:00-4:59 PM	14 (8.1)	5 (5.5)	9 (11.0)
	5:00-9:00 PM	24 (13.9)	9 (9.9)	15 (18.3)
	Does not matter	59 (34.1)	35 (38.5)	24 (29.3)
**Preferred frequency of receiving messages (n=171)**				
	≥Twice/day	3 (1.8)	2 (2.2)	1 (1.3)
	Once/day	42 (24.6)	31 (34.1)	11 (13.8)
	5-6 times/week	8 (4.7)	6 (6.6)	2 (2.5)
	3-4 times/week	17 (9.9)	9 (9.9)	8 (10.0)
	1-2 times/week	68 (39.8)	29 (31.9)	39 (48.8)
	1-3 times/month	33 (19.3)	14 (15.4)	19 (23.8)
**Receiving messages in (n=170)**				
	One-way only	59 (34.7)	46 (50.5)	13 (16.5)
	Two-way	111 (65.3)	45 (49.5)	66 (83.5)
Satisfaction toward the program (n=172)		153 (89.0)	75 (82.4)	78 (96.3)

The overall satisfaction rate with the program was 89.0 % (see Table 4). Participants rated this intervention to be helpful with improving adherence to medications (80.9 %), dietary recommendations (76.3%), increased physical activity (66.5%), and smoking cessation (60.9 %). The majority of patients perceived that the app improved physician-patient communication (74.6%).

We also received suggestions from physicians and patients on how to improve the intervention. Major suggestions included the following: incorporation of the intervention within the current health information system, further customizing message content to individual conditions, and talking slower on the recorded phone call.

A total of 11,534 messages were successfully delivered; however, because of local regulation policy, 166 messages failed to be sent at the beginning stage. All voice calls (720 in total) were successfully dialed and each voice call lasted for 1 to 2 min. During the course of the study, no patient requested cessation of message delivery.

The average cost charged by the carrier was approximately 0.10 RMB (around US $0.015) per text message and 0.09 RMB (around US $0.013) per voice call. To complete the 12-week intervention, it was estimated that for smokers, the average cost was 6 RMB (US $0.9) for the text-message program and 5.4 RMB (US $0.8) for the voice-call program; for nonsmokers, the costs were 4.8 RMB (around US $0.74) and 4.3 RMB (around US $0.66), respectively.

## Discussion

### Summary of Findings

To our knowledge, this is the first mixed-method study to target both providers and patients in China using mHealth technology to improve the secondary prevention of CHD. The intervention was feasible with easy-to-use products, low operational costs, and well accepted by patients, though the use of app prescription function was challenging for some physicians. We found that a multifaceted intervention that included a provider-facing Android app and a 12-week program of text messages or phone calls directed at patients with CHD was associated with an improvement in patient medication adherence (Hainan), smoking cessation (combined), and vegetable consumption (Shanghai) with notable regional differences that merit additional investigation.

### Use of Mobile Health Technology

The ubiquity of mobile phones, especially in resource-limited settings, has made mHealth interventions an attractive option to modify health-related behaviors [25]. Quite a few studies have been conducted to improve the secondary prevention of CHD patients through text messages, and the evidence available seems consistent in supporting the positive effect of the intervention on medication adherence [26-28] and some have demonstrated the effectiveness on smoking cessation [29] and dietary habits [27,28].

In this study, we designed a mobile app for physicians and text messages for patients with the ultimate objective to improve medication adherence. Our working mechanism is to create a new follow-up platform through which patients could receive evidence-based support and recommendations from physicians to increase their compliance and improve their lifestyle.

### Interpretation of the Findings

Interestingly, we found an association between the intervention and medication adherence among Hainan patients only. Possible drivers of the site-specific effect were differences in the underlying population, including burden of comorbidities, saturation of secondary prevention knowledge among patients, and hospitalization effect. The greater severity of heart disease conditions among patients recruited in Hainan (reflected in their MI proportion and medications number in Table 1) may account for the bigger changes in medication adherence, given that these patients likely had stronger motivations to follow treatment recommendations. We also observed from the patient interviews that the saturation of secondary knowledge among patients was lower, as they could only access secondary prevention knowledge through hospital physicians and this study. Four patients interviewed in Hainan claimed that this study was their only source for information regarding secondary prevention management. Shanghai patients, in comparison, could access the information through primary care providers, community health lectures, and family members. This would suggest that the benefit of messaging-based interventions might be greatest in low-resource settings with a dearth of reliable sources for health-related information. In addition, patients in Hainan experienced recent hospitalization, which might trigger them to better conform to physicians. Due to lack of control arm, we could not exclude the possibility that it was the recent hospitalization that provoked the change, which needs to be further verified in future studies. Though we found differences in patient age, hypertension history, heart diseases type, and time since diagnosis of heart disease between the two sites, the results suggest that those were not the driving factors of the difference in medication adherence.

The study results showed that the intervention might correlate with the improvement in smoking cessation, which is consistent with other mHealth trials [10,29-31]. We observed the association between intervention and the improvements in dietary habits, though it was unclear why the change of vegetables consumption was obvious in Shanghai only. The visit frequency to the community health center in Shanghai increased from 7 to 10 in 3 months. To regulate overprescribing behaviors of physicians in China, the essential medicines policy restricts that physicians in primary health care centers can only prescribe medicines at 1-month dosage. The increased frequency is a positive sign that the intervention promotes the physician-patient communication, and patients tended to have more frequent visit to the community health care centers.

### Barriers Encountered During Implementation

Despite the solid mobile technology infrastructure in China, we encountered some technical barriers at implementation. Our text messages were initially suspended by the carrier for 3 to 5 days after pilot testing, as they were mistaken as fraud or commercial messages because of the high volume of messages. This was quickly rectified after we contacted the customer service departments of the telecommunications carrier and explained the study protocol. For some patients who chose to receive messages by phone call, the caller ID was not displayed correctly so the patients tended to incorrectly identify them as unsolicited advertising calls and hung up. This issue should be addressed in future studies by assigning a unique caller ID for incoming calls that would be shared with patients at enrollment.

The use of the app prescription function was partially restricted by the current Chinese health system. In the community health center, the function was limited as a result of the newly implemented essential medicine policy. The policy made a list of 307 *essential medicines* that could be stored at primary care centers and sold at low prices in 2009 and updated in 2012 to extend it to 520 medicines (each province can add medicines based on specific contexts) [32,33]. However, it turns out that it indirectly restricts the prescription rights of primary care providers as many truly essential medicines are not on the list. Therefore, some of the medicines suggested by the NICE guidelines, such as clopidogrel, could not be prescribed. Physicians in tertiary hospitals have full prescription rights; however, their heavy workload spared them little time to use the app.

### Strengths and Limitations of the Study

Our study shows that the intervention is generally feasible. Our study targets both physicians and patients using technology-enabled intervention. In particular, the use of two disparate test sites—a community health center and a tertiary care hospital—greatly enhance the generalizability of our findings within the Chinese health care system. Differences in the findings from these two sites provided rich insights for future implementation research. Our understanding of the results was enhanced by the use of the mixed-method approach.

A principal limitation is that this study does not have a control group, which increases the risk that the change may not be attributed to the intervention only, although we built a regression model to control for possible confounders to mitigate the risk. Future studies with control arm are needed to evaluate its effectiveness in resource-limited settings. The small sample size and relatively short follow-up may preclude detection of small changes in adherence and lifestyle modifications and evaluation of persistence of efficacy over time. The self-reported nature of the outcomes means that results may be contaminated by recall or social likeability biases. Future studies may consider more objective measures, including pill counting for medication adherence, physiologic assessments (blood pressure, cholesterol, and glucose control), and clinical outcomes (MI, revascularization, and death). It may be necessary to include additional confounding variables, such as socioeconomic and psychosocial status, to sufficiently demonstrate the effects. Moreover, future studies should further examine fidelity to the intervention, that is, how many people actually read the messages or listened to the calls to accurately reflect their adherence to the intervention. Future studies could also record data related to number of eligible patients and reasons for nonparticipation to enhance the transparency of the recruitment process. Finally, the intervention was not incorporated within the current workflow of Chinese physicians, which was a barrier to its rapid and widespread adoption. Despite these limitations, this study provides experiences of implementing the TAKEmeds intervention model, its feasibility, and preliminary effectiveness results in two different settings in China.

### Conclusions

Both international and national guidelines targeting medicine use and lifestyle modifications are well established to improve the secondary prevention of CHD; however, its uptake into clinical practice is far from optimal in China and around the world.

Such challenges require innovative, feasible, and cost-effective solutions. Harnessing the ubiquity of mobile phones and rapid advance in low-cost mobile technology, this study devised a multifaceted intervention package that targets both providers and physicians to increase the uptake of evidence-based secondary prevention of CHD in China. The results support the feasibility of the intervention: easy to use; well accepted by patients; and showing potential effect in improving medication adherence, smoking status, and possibly vegetables consumption as well as physician-patient interaction in community health centers, though we believe future studies with control group are needed to verify it before scaling up this intervention. We further found that the TAKEmeds intervention might have greater potential to improve the outcomes in resource-limited places such as Hainan, and further studies with control arms should verify the impact of the mHealth intervention across various economical settings. Although this study has several limitations, it provides a proof of concept for the role of mHealth in cardiovascular prevention. If these findings are confirmed in future studies with control arm and clinical endpoints, the intervention can be scaled up across the health system in China and other low-resource settings and can be adapted to other chronic diseases such as hypertension and diabetes.

## Appendix

Multimedia Appendix 1 Supplemental file (tables).

## Abbreviations

ACE: angiotensin-converting enzyme inhibitors.
ARB: angiotensin receptor blockers.
CHD: coronary heart disease
GDP: gross domestic product
HPNGH: Hainan Provincial Nongken General Hospital
MET: Metabolic Equivalent
mHealth: mobile health
MI: myocardial infarction
NICE: National Institute for Health and Care Excellence
OR: odds ratio
RMB: Ren Min Bi (Chinese Currency)
SMS: short message service
TAKEmeds: the Adherence and Knowledge Exchange heart disease medicines study
