# Supplementary material for: Using Mobile Health Intervention to Improve Secondary Prevention of Coronary Heart Diseases in China: Mixed-Methods Feasibility Study
Source: JMIR Mhealth Uhealth. 2018 Jan 25;6(1):e9. doi: 10.2196/mhealth.7849 (PMC5806005; doi:10.2196/mhealth.7849)
Supplement: Multimedia Appendix 1 [file mhealth_v6i1e9_app1.pdf]

Table 1. Full model results

	Odds ratio	SE	Z	P>|z|	95% CI	
Time	1.80	0.42	2.51	0.012	1.14	2.85
Region	1.73	0.54	1.76	0.079	0.94	3.21
Age <50	1.20	0.73	0.30	0.765	0.36	3.95
Age 50-59	1.48	0.89	0.66	0.509	0.46	4.78
Age 60-69	1.86	1.13	1.02	0.307	0.56	6.14
Age 70-79	1.92	1.34	0.94	0.350	0.49	7.51
Diagnosed 1 year ago	0.87	0.25	-0.47	0.639	0.49	1.54
Hypertension	1.15	0.33	0.49	0.624	0.65	2.03
Diabetes	1.03	0.30	0.10	0.921	0.58	1.83
Took 2 medicines	3.11	1.50	2.35	0.019	1.21	8.01
Took 3 or more medicines	2.66	1.21	2.15	0.031	1.09	6.50

Table 2. Model results with region as interaction term

	Odds ratio	SE	Z	P>|z|	95% CI	
Time	0.61	0.19	-1.58	0.114	0.33	1.12
Region	0.57	0.23	-1.39	0.163	0.26	1.26
TimeXregion	23.93	14.83	5.12	0.000	7.10	80.61
Age <50	1.23	0.88	0.29	0.770	0.30	4.98
Age 50-59	1.66	1.16	0.72	0.470	0.42	6.52
Age 60-69	2.14	1.52	1.07	0.285	0.53	8.61
Age 70-79	2.25	1.81	1.00	0.315	0.46	10.91
Diagnosed 1 year ago	0.86	0.29	-0.44	0.660	0.45	1.65
Hypertension	1.23	0.41	0.64	0.522	0.65	2.35
Diabetes	0.97	0.32	-0.10	0.924	0.51	1.86
Took 2 medicines	3.33	1.81	2.21	0.027	1.15	9.69
Took 3 or more medicines	2.74	1.41	1.96	0.050	1.00	7.50

Time+TimeXRegion	14.68	7.77	5.07	0.000	5.20	41.45
